# Efficacy of Keratin AE1/AE3 Staining in Evaluating Resection Margins of a Urachal Remnant and Improving Surgical Precision and Outcomes: A Case Report

**DOI:** 10.7759/cureus.63197

**Published:** 2024-06-26

**Authors:** Kyoko Baba, Ami Kuwabara

**Affiliations:** 1 Department of Plastic and Aesthetic Surgery, Kitasato University School of Medicine, Sagamihara, JPN; 2 Department of Plastic Surgery, Kitasato University Medical Center, Kitamoto, JPN

**Keywords:** surgical resection, urachal epithelium, keratin ae1/ae, immunohistological staining, margin evaluation, urachal remnant

## Abstract

A urachal remnant is a rare condition characterized by the persistence of the urachus beyond birth, often presenting with symptoms such as umbilical effusion, periomphalitis, and abdominal pain. Surgical resection is the cornerstone of treatment, but ensuring complete removal of urachal epithelium at the resection margin remains a challenge. This case report focuses on evaluating resection margins of urachal remnants and reports the case of a 25-year-old woman with complaints of umbilical effusion and a mass. She was diagnosed with a urachal remnant and underwent urachal resection and reconstruction, with postoperative confirmation of favorable outcomes and the absence of microscopic hematuria. The intraoperative examination did not reveal any macroscopically clear luminal structure of the urachal resection margin. Subsequent histopathological analysis of the margin using hematoxylin and eosin staining was challenging, prompting the use of immunohistological staining with keratin AE1/AE3 antibody. The antibody did not stain the urachal resection margin, confirming the complete removal of urachal epithelial components. Our study findings suggest the utility of keratin AE1/AE3 staining for assessing urachal remnant margins and underscore the importance of thorough evaluation and complete resection of urachal remnant to prevent recurrence and mitigate the risk of urachal cancer, contributing to improved surgical outcomes and patient care.

## Introduction

A urachal remnant is characterized by the persistence of the urachus, a fetal structure located between the bladder and the umbilicus, after birth, and presents with symptoms such as urinary leakage from the umbilical site, periomphalitis, and abdominal pain [1,2]. These symptoms may manifest not only during the neonatal period and childhood but also during adolescence and adulthood [3]. At our department, surgical intervention is typically warranted for urachal remnant patients exhibiting symptoms after adolescence, particularly those with lesions localized between the umbilical fossa and the abdominal wall, and without concurrent urological issues. While cases of urachal cancer have been reported previously [4-7], the condition is rare, underscoring the importance of ensuring the complete removal of urachal epithelium during surgery to mitigate the risk of cancer. While histopathological examination has traditionally been utilized to assess residual urachal epithelium at the resection margin, limitations have been observed, with instances where definitive exclusion of urachal epithelial cells using hematoxylin and eosin (HE) staining alone has been challenging.

To our knowledge, no study to date has reported immunohistochemical staining for evaluating the urachal remnant margins. We proposed the use of immunohistochemical staining of epithelial tissues at the resection margin to more accurately evaluate residual urachal epithelium. Specifically, we focused on keratin AE1/AE3, known for its high utility in clinical practice. Here, we report a case of urachal remnant where we demonstrate the utility of immunohistological staining with keratin AE1/AE3 antibody in evaluating the resection margin, addressing the challenges encountered with conventional histopathological examination and providing valuable insights for improving surgical management and outcomes through our novel approach. This is the first report focusing on the evaluation of the urachal remnant margin using this method.

## Case presentation

**Figure 1:**
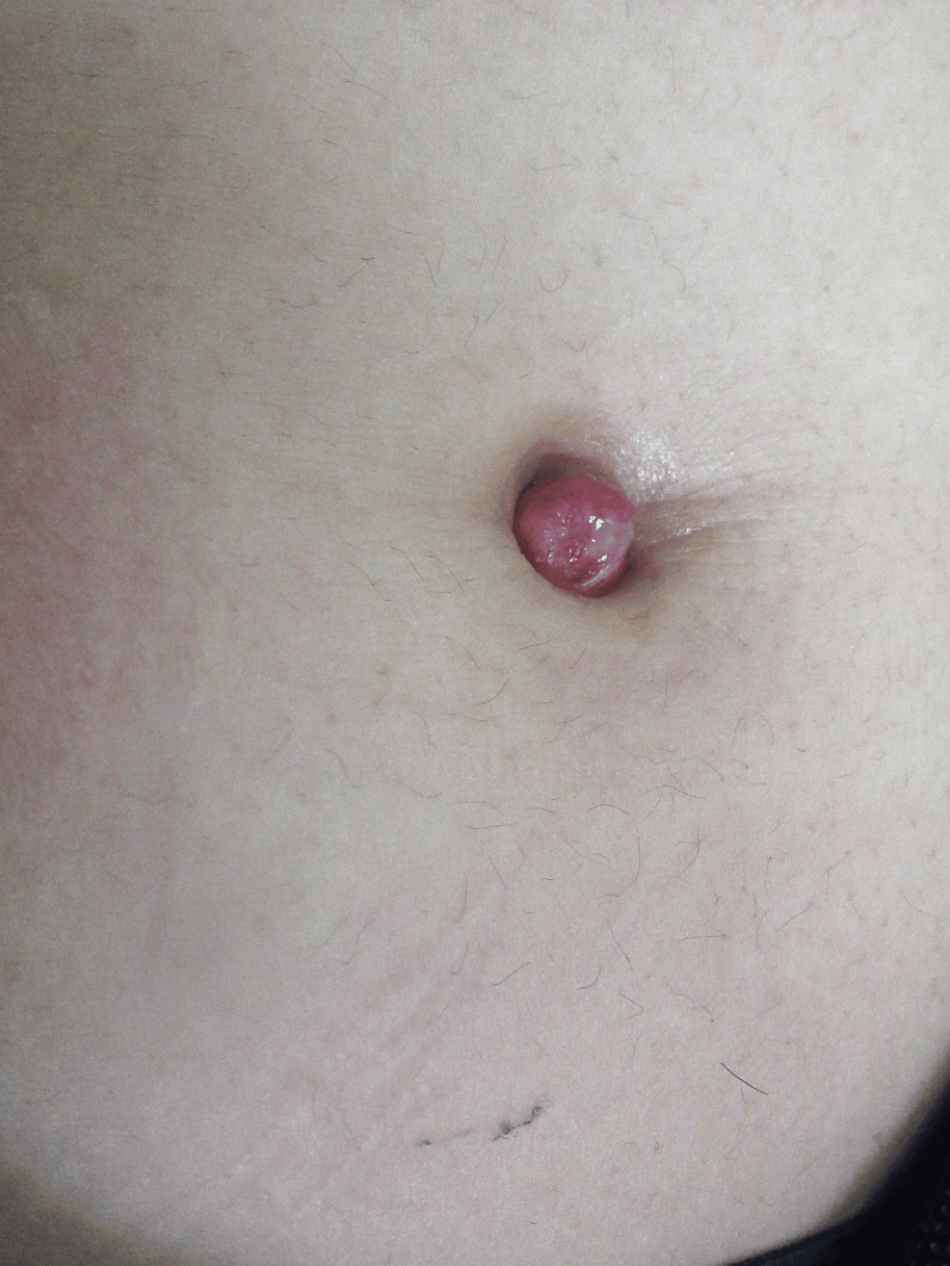
Findings at the first visit. Findings at the first visit to our facility showing poor granulation with effusion observed in the umbilical fossa.

A 25-year-old woman presented with chief complaints of umbilical effusion and an umbilical mass. Her past medical history was unremarkable. Approximately one month before her initial visit to our department, she sought medical attention from a local physician, who subsequently referred her to the surgery department of our hospital and then to our department. Upon examination at her initial visit, poor granulation with effusion was observed in the umbilical fossa (Figure 1).

There were no symptoms of peritoneal irritation, and blood tests revealed no abnormal findings, including an absence of increased inflammatory response. Further evaluation by the urology department, including urinalysis and cystoscopy, showed no significant findings.

**Figure 2:**
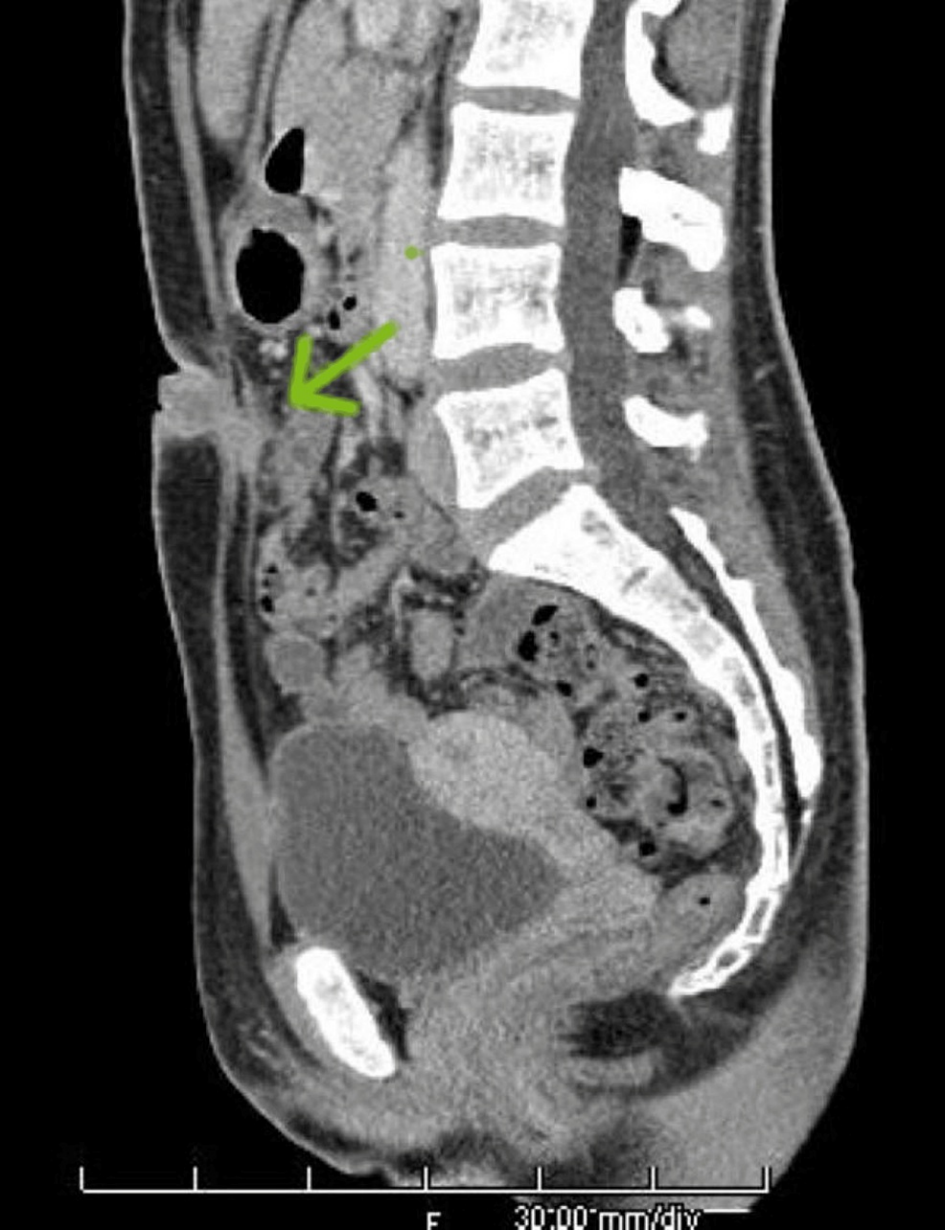
Contrast CT findings at the first visit: sagittal section. The arrow indicates the urachal remnant of the umbilical urachal sinus type and internal poor granulation.

Contrast CT scan revealed a tubular structure extending from immediately below the umbilical fossa to the midline of the abdominal wall, indicative of a urachal remnant of the umbilical urachal sinus type (Figure 2).

Surgery was performed following the resolution of inflammation at the umbilical site, achieved through oral administration of an antibacterial agent (cefcapene pivoxil hydrochloride hydrate 300 mg/day) and topical application of povidone-iodine gel. Urachal resection and umbilical reconstruction were performed under general anesthesia. A spindle-shaped incision was made in the skin within the umbilical fossa, encompassing the opening, followed by the removal of scar-like subcutaneous tissue of the umbilical fossa around the area. Subsequently, an incision was made caudal to the subumbilical midline, approximately 2 cm from the spindle-shaped incision in the umbilical fossa. After confirming the preperitoneal adipose tissue, the abdomen was opened from the cranial side while paying attention to intraperitoneal adhesions. A surgical probe was inserted through the skin opening, and after macroscopically confirming it from the back of the abdominal wall, the urachus located in the midline was identified. As we pulled the urachus along with the skin to be resected together, its removal proceeded toward the bladder. The left and right lateral umbilical artery cords were ligated and resected. The urachus was double ligated and resected at approximately 1 cm caudal to the site where there was a macroscopic luminal structure and loss of residual inflammation findings, such as dilation and proliferation of peritoneal capillaries. Approximately 9 cm of the urachus was removed from the skin opening at the umbilical fossa. The abdomen was closed after confirming the absence of ascites fluid accumulation in Douglas’s pouch. The skin of the umbilical fossa was attached to the linea alba of the closed abdomen, and umbilical reconstruction was performed.

**Figure 3:**
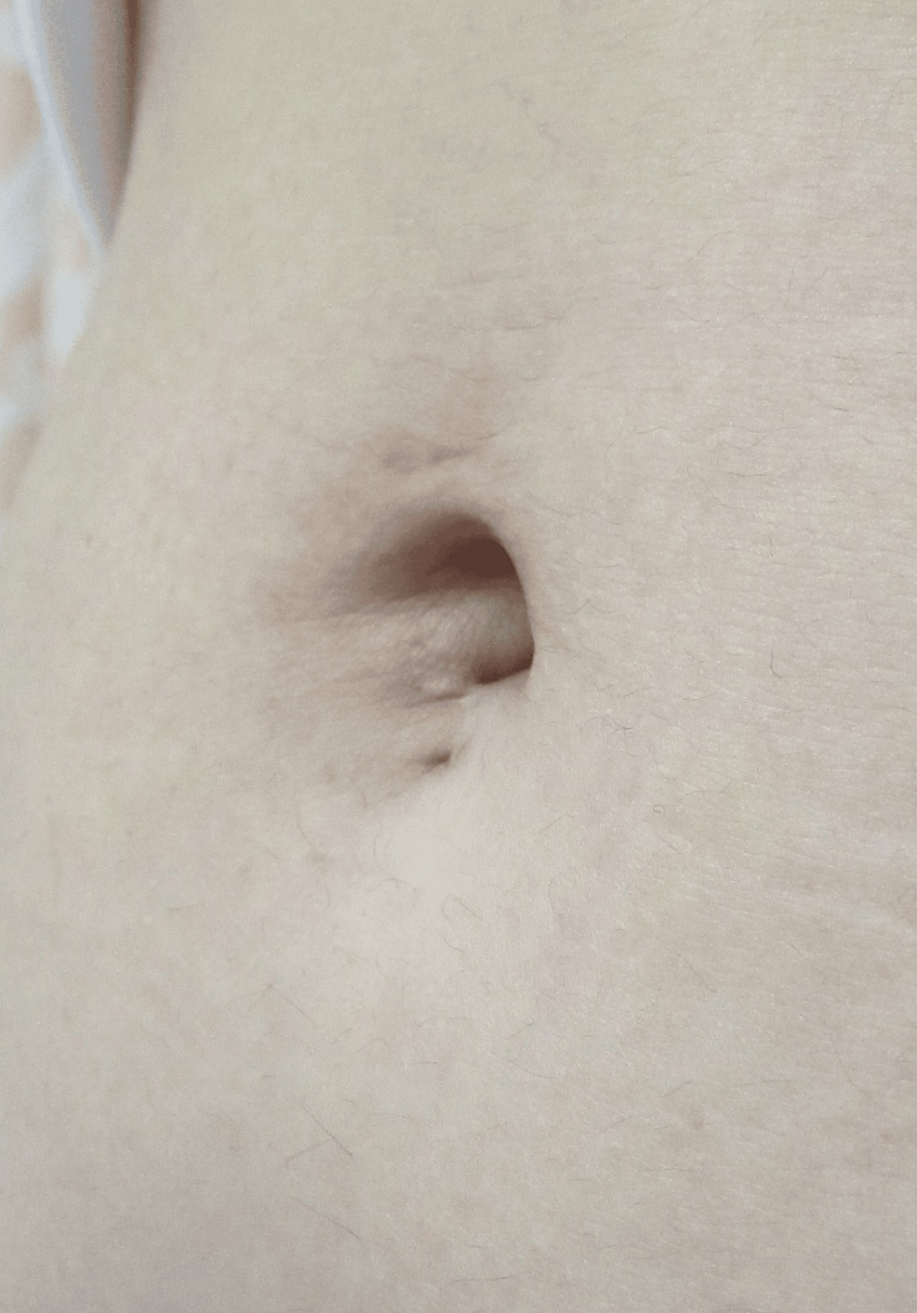
Findings in a standing position at postoperative month three. The morphology of the umbilical fossa was favorable. The scar was mostly within the umbilical fossa, and the subumbilical scar was not prominent.

The absence of microscopic hematuria was confirmed on postoperative day two, and after the removal of the urethral catheter, the patient was discharged from the hospital on postoperative day four. She was followed up as an outpatient, and the postoperative course and umbilicus morphology were favorable, indicating a satisfactory outcome (Figure 3).

**Figure 4:**
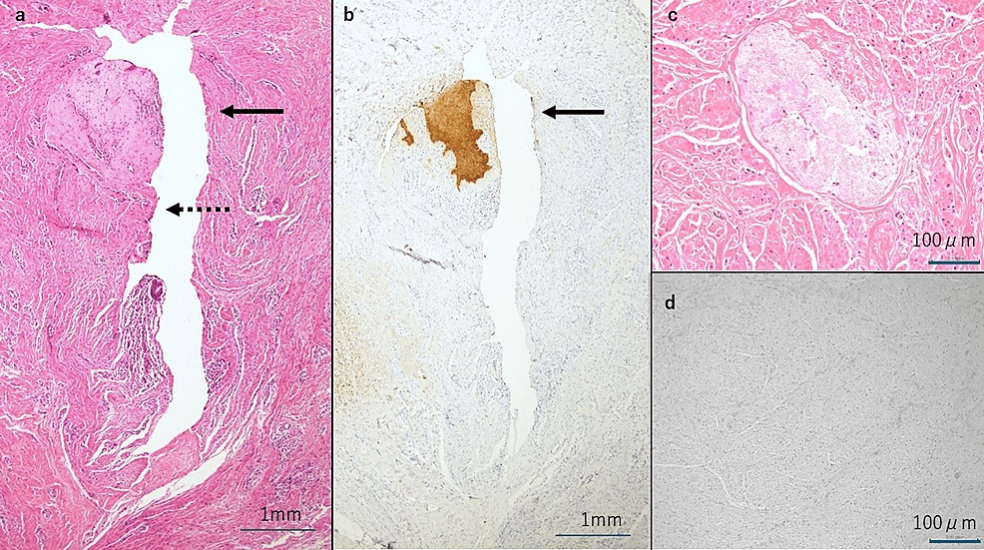
Histopathological and immunohistological findings. (a) Hematoxylin and eosin (HE) staining (longitudinal section): The solid arrow indicates the skin, and the dotted arrow indicates the urachal lumen. A luminal structure continuous from the skin was observed, suggestive of a urachal remnant. (b) Keratin AE1/AE3 staining (longitudinal section): The solid arrow indicates the skin. The skin was stained with keratin AE1/AE3 antibody. The cells of the umbilical side were positive for keratin AE1/AE3 staining, while the bladder side was negative. The urachal epithelial cells were not identified at the resection margin. (c) HE staining of the cross-section near the distal resection margin: The luminal structure was unclear. (d) Keratin AE1/AE3 staining of the cross-section near the distal resection margin: No cells were stained with keratin AE1/AE3 antibody. The urachal epithelial cells were not identified at the resection margin.

On histopathological examination, HE staining revealed a luminal structure continuous from the skin, suggestive of a urachal remnant. However, no clear luminal structure was observed at the distal margin (Figures 4a, 4c). Immunohistological staining with keratin AE1/AE3 antibodies showed positive staining on the umbilical side of the tissue facing the luminal structure. Conversely, no staining was observed in multiple sites closer to the bladder or near the resection margin, confirming the absence of urachal epithelium in the margin (Figures 4b, 4d).

## Discussion

This study reports a case demonstrating the efficacy of immunohistological staining with keratin AE1/AE3 antibody for evaluating urachal remnant margins, revealing its superior reliability compared to traditional HE staining.

During fetal development, the urachus, derived from the allantoic membrane, lies between the bladder and the umbilical cord, gradually closing by the fourth to fifth month of intrauterine life to form the median umbilical ligament in adulthood [8]. However, urachal remnant presents when the urachus fails to close after birth [1,2,9], manifesting symptoms such as urinary leakage from the umbilical site, periomphalitis, abdominal pain, infection in the umbilical fossa, and a subumbilical mass [1,3,10]. Additionally, cases have been reported in which the urachus that had closed after birth reopened due to infection, increased intravesical pressure, or other causes, resulting in the development of the disease [3]. While the prevalence of urachal remnant is significant, with 50% in fetuses immediately before birth and 2% in adults [3,8], diagnosis remains challenging due to varied clinical presentations. MRI is considered useful for its diagnosis, and its morphological classification has been presented by Blichert-Toft et al. [10], which classifies a urachal remnant into the following five types: (A) congenital patent urachus, (B) umbilical urachal sinus, (C) vesicourachal diverticulum, (D) urachal cyst, and (E) the alternating sinus (forming a fistula due to infected urachal cyst). The incidence rates have been estimated to be 15% for patent urachus, 49% for urachal sinus, and 36% for urachal cyst [10]. Nevertheless, the clinical classification of urachal remnant is difficult [11]. The treatment provided at our department targets patients developing the urachal sinus type (type B in Blichert-Toft classification) after adolescence, with a lesion in the abdominal wall.

The recurrence of inflammation has been estimated at 30% in patients who had developed an infected urachal remnant and undergone incision and drainage without extirpation [3]. In addition, although urachal cancer is rare, accounting for only approximately 0.4% of all urological malignant diseases, the potential risk of its development should also be considered [12]. Because the histological types of urachal cancer in most cases reported thus far are adenocarcinoma and transitional cell carcinoma, complete resection of epithelial components is thought to eliminate the cause of urachal cancer development [13]. Consequently, surgical resection remains the cornerstone treatment for urachal remnants. When managing patients with urachal remnant infection, extirpation is recommended after controlling the infection through incision or other means, aiming to minimize the risk of surgical site infection after extirpation. Additionally, in plastic surgery, umbilical reconstruction is commonly performed during urachal resection [14].

At our hospital, the treatment approach for urachal remnant involves collaborative efforts with the urology department before surgery. This collaboration serves two main purposes: first, to assess the feasibility of completing the surgery by manipulating the abdominal wall, and, second, to ensure thorough removal of epithelial components from the urachal lumen during the procedure. Additionally, our team endeavors to minimize invasiveness and scarring by opting for small laparotomy and strategically placing the scar within the umbilical fossa during umbilical reconstruction.

For histopathological evaluation of urachal remnant, urachal epithelial cells can be easily identified by HE staining if the luminal structure is clear. However, at sites with a small lumen, identification of urachal epithelial cells is difficult with this type of staining alone. Previously, when managing cases with a short distance between luminal structure loss and the margin, uncertainty persisted regarding the presence of residual urachal epithelial cells despite the absence of observable luminal structures. Thus, to determine the presence or absence of urachal epithelial cells in our case, we evaluated the margin by immunohistological staining. Using keratin AE1/AE3 antibody, known for its superior staining capacity, we were able to conclusively determine the absence of residual urachal epithelial cells more reliably than with HE staining alone. The epithelial cocktail antibody of keratin AE1/AE3 is highly versatile, with AE1 and AE3 reacting with type I and II cytokeratin, respectively [15].

This staining does not provide intraoperative assistance. Nevertheless, this staining helps to confirm whether the urachal epithelial cells, which are the origin of urachal carcinoma, have been completely resected. We recommend utilizing this staining for postoperative follow-up. If residual urachal epithelial cells are present, careful postoperative monitoring should be conducted.

## Conclusions

Because the histological types of urachal cancer in most cases reported thus far are adenocarcinoma and transitional cell carcinoma, complete resection of epithelial components is important. This is the first report focusing on the evaluation of the urachal remnant margin. This case demonstrated the efficacy of the combined use of keratin AE1/AE3 staining in determining the absence of residual urachal epithelial cells easily and more reliably than HE staining alone. Our findings suggest the usefulness of keratin AE1/AE3 staining in the evaluation of residual urachal epithelium at resection margins of a urachal remnant.
